# Leptin and inflammatory pathways in the Alzheimer's disease continuum: Implications for glial activation and neuropsychiatric symptoms

**DOI:** 10.1016/j.bbih.2025.101018

**Published:** 2025-05-26

**Authors:** Fumihiko Yasuno, Kazuyuki Nakagome, Yoshie Omachi, Yasuyuki Kimura, Aya Ogata, Hiroshi Ikenuma, Junichiro Abe, Hiroyuki Minami, Takashi Nihashi, Kastunori Yokoi, Saori Hattori, Nobuyoshi Shimoda, Kensaku Kasuga, Takeshi Ikeuchi, Akinori Takeda, Takashi Sakurai, Kengo Ito, Takashi Kato

**Affiliations:** a National Hospital for Geriatric Medicine, National Center for Geriatrics and Gerontology, Obu, Japan; b Department of Clinical and Experimental Neuroimaging, Center for Development of Advanced Medicine for Dementia, National Center for Geriatrics and Gerontology, Obu, Japan; c Department of Psychiatry, National Center of Neurology and Psychiatry, Kodaira, Japan; d Department of Pharmacy, Faculty of Pharmacy, Gifu University of Medical Science, Kani, Japan; e Functional Genomics Unit, Medical Genome Center, National Center for Geriatrics and Gerontology, Obu, Japan; f Department of Molecular Genetics, Brain Research Institute, Niigata University, Niigata, Japan

**Keywords:** Alzheimer's disease, Leptin, Adiposity, Neuropsychiatric symptoms, Positron emission tomography, Neuroinflammation, Insula

## Abstract

**Introduction:**

Chronic peripheral inflammation triggered by adipokine release may extend to the brain, potentially influencing the pathological progression of Alzheimer's disease (AD) and neuropsychiatric symptoms (NPS). However, it remains unclear whether and how leptin contributes to the link between adipose tissue dysfunction and dementia. This study aims to investigate the role of leptin in the connection between adipose-derived inflammatory signaling and cognitive impairment/NPS.

**Methods:**

Path analysis was employed to explore how leptin relates to the association between adipose-related metabolic dysfunction and dementia through inflammatory pathways in patients with AD pathology (n = 15). Variables included plasma leptin concentration, body mass index (BMI) as a marker of adiposity, and in vivo assessments of regional neuroinflammation using translocator protein (TSPO)-PET imaging with the tracer ^11^C-DPA-713 (^11^C-DPA-713-binding potential [^11^C-DPA-713-BP_ND_]). Cognitive function was measured using the Alzheimer's Disease Assessment Scale-Japanese Cognitive Subscale (ADAS-J cog), while NPS were assessed using the Neuropsychiatric Inventory Questionnaire (NPI-Q).

**Results:**

Regression analysis demonstrated that higher plasma leptin concentrations positively correlated with BMI and significantly predicted ^11^C-DPA-713-BP_ND_ in the insula. Additionally, NPI-Q scores were associated with ^11^C-DPA-713-BP_ND_ in the insula. Path analysis supported leptin's role linking adiposity to NPS through insular inflammation. The hypothesized model fit the data well under the null hypothesis [χ^2^ (3) = 0.63, p = 0.89].

**Discussion:**

These findings underscore the relevance of exploring how leptin and adipose tissue dysfunction interact with neuroinflammatory processes in contributing to NPS in the patients in the AD continuum. Interventions targeting these interactions could represent promising avenues for managing NPS.

## Highlights

• Used path analysis to explore leptin's role linking adiposity and dementia.
• Leptin links adiposity to neuropsychiatric symptoms via insular inflammation.
• Leptin-targeted interventions may offer an option for neuropsychiatric symptoms in AD.

## 1 Introduction

Alzheimer's disease (AD) is the most common form of dementia, characterized by two primary brain abnormalities: senile plaques mainly made up of amyloid-beta (Aβ) peptides and neurofibrillary tangles predominantly formed by highly phosphorylated tau protein (p-tau). The disease often begins with episodic memory deficits associated with hippocampal damage. In addition to cognitive deterioration, nearly all patients with AD experience neuropsychiatric symptoms (NPS) (Canevelli et al., 2013; Cummings et al., 2019; Deardorff and Grossberg, 2019). Furthermore, various adipose-related pathological changes, including neuroinflammation, insulin resistance, and mitochondrial dysfunction, have been observed in the progression of AD (O'Brien et al., 2017)**.**

Dysregulated adipose tissue function leads to the secretion of soluble mediators known as adipokines, which modulate immune responses and energy homeostasis. Leptin is one such adipokines and has been recognized as a critical regulator in controlling food consumption and body weight (Zhang et al., 1994). Circulating leptin levels reflect the body's energy reserves in adipose tissue and are closely associated with the total body fat mass. Typically, individuals with higher adiposity produce higher levels of leptin compared to those with lower adiposity (Considine et al., 1996; Frederich et al., 1995; Maffei et al., 1995). Leptin has many other functions besides controlling food intake: it is a growth factor, promotes puberty, regulates metabolism, and is involved in memory. These effects are facilitated by the interaction of leptin with its dedicated receptors found in both the central nervous system (CNS) and peripheral tissues (Kelesidis et al., 2010; Margetic et al., 2002; McGregor and Harvey, 2018).

Leptin is essential for the regulation of both innate and adaptive immune responses. Elevated leptin levels disrupt homeostasis and are linked to the onset of multiple disorders, including neurodegenerative conditions. This may be due to leptin's structural similarity to the long-chain helical cytokine family, which encompasses interleukin (IL)-6, IL-11, IL-12, and oncostatin M (La Cava and Matarese, 2004). Leptin connects nutritional status to T helper 1 immune responses through its immune-modulating effects, and lower plasma leptin levels are frequently linked to weakened immune function (Lord et al., 1998). Chronic peripheral inflammation induced by adipokine release has the potential to spread to the brain, suggesting that leptin might influence the pathological progression of AD and neuropsychiatric symptoms (Pérez-Pérez et al., 2020). However, it remains unclear whether and how leptin contributes to the link between adiposity and dementia through the induction of inflammation.

Positron emission tomography (PET) has become an important method for visualizing and measuring activated glial cells in the brain, offering insights into how neuroinflammation influences disease progression. The 18-kDa translocator protein (TSPO) acts as a biomarker for assessing the level of inflammatory response (Banati et al., 2004). Under pathological conditions, TSPO levels rise in response to glial activation, and administering a radiolabeled tracer that binds to TSPO allows for in vivo PET imaging of glial cell-mediated neuroinflammation. The TSPO antagonist 11C-N,N-diethyl-2(2-(4-methoxyphenyl)-5,7-dimethyl-pyrazolo[1,5-α]pyrimidin-3-yl)-acetamide (^11^C-DPA-713) has been developed, featuring high target binding affinity and low lipophilicity (Boutin et al., 2007). PET quantification of TSPO binding of ^11^C-DPA-713 can serve as a sensitive method to assess the extent of glial activation caused by neuroinflammation.

This study employed path analysis to investigate the role of leptin in linking adiposity to dementia through inflammatory pathways in the AD continuum. Variables analyzed included plasma leptin levels, body mass index (BMI) as an indicator of adiposity, and regional neuroinflammation measured in vivo through TSPO-PET imaging with the tracer ^11^C-DPA-713 (binding potential of ^11^C-DPA-713 [^11^C-DPA-713-BP_ND_]). Cognitive function was assessed using the Alzheimer's Disease Assessment Scale-Japanese Cognitive Subscale (ADAS-J cog), and NPS were measured with the Neuropsychiatric Inventory Questionnaire (NPI-Q) (Kaufer et al., 2000). We hypothesized that a) patients in the AD continuum with higher BMI would exhibit higher plasma leptin concentration; b) a higher plasma leptin concentration would be related to higher regional neuroinflammation which was associated with the cognitive deterioration and/or NPS along the AD continuum.

## 2 Material and methods

### 2.1 Participants

This cross-sectional study included fifteen patients in the AD spectrum, comprising eight individuals with mild cognitive impairment (MCI) and seven with AD, all of whom had data on plasma leptin levels and TSPO-PET imaging. These participants showed positive amyloid-β/p-tau 181 markers in cerebrospinal fluid (CSF) analysis. Inclusion criteria for patients with MCI were a Mini-Mental State Examination (MMSE) score between 24 and 30, confirmed objective memory impairment, a Clinical Dementia Rating (CDR) of 0.5, and the ability to independently perform basic daily activities. The diagnosis of probable AD was made according to clinical guidelines established by the National Institute on Aging and the Alzheimer's Association (McKhann et al., 2011), with an MMSE score below 24. According to the rs6971 polymorphism in TSPO, all patients were categorized as exhibiting high binding affinity (Owen et al., 2011).

Patient CSF samples were preserved at −80 °C in the biobank at the National Center for Geriatrics and Gerontology, Japan. Amyloid and p-tau 181 positivity were assessed by measuring the CSF Aβ42/40 ratio and p-tau 181 levels, respectively, at the Brain Research Institute of Niigata University, Japan. The thresholds for defining amyloid and p-tau 181 positivity were set at <0.072 (Kasuga et al., 2023) for amyloid and >30.6 pg/mL (Kasuga et al., 2022) for p-tau 181, based on CSF samples obtained from participants in the Japanese Alzheimer's Disease Neuroimaging Initiative.

This study was approved by the Institutional Review Board of the National Center for Geriatrics and Gerontology. Written informed consent was obtained from all participants before their involvement in the research.

### 2.2 Plasma biomarker assay

For plasma extraction, 24 ml of whole blood was drawn from each patient into ethylenediaminetetraacetic acid (EDTA) tubes. The blood samples were then centrifuged at 2350 g for 5 min at 4 °C. Post-centrifugation, 0.3 ml of plasma was aliquoted into 1.5-ml tubes and stored at −80 °C in the NCGG biobank.

Plasma concentrations of C-reactive protein (CRP), IL-1β, IL-6, leptin, and tumor necrosis factor-alpha (TNF-α) were measured at Acel, Inc. (Tokyo, Japan), using the Luminex Human Discovery Assay (R&D Systems, Inc., #LXSAHM-07) following the manufacturer's guidelines on a Bio-Plex 200 system (Bio-Rad Laboratories, Inc.) with low photomultiplier tube settings. Analyte levels were calculated using Bio-Plex Manager software (version 6.2.0.175, Bio-Rad Laboratories, Inc.).

### 2.3 Neuropsychiatric symptoms assessment

The severity of NPS was assessed using the 12-item NPI-Q, with scores from 0 to 3, with higher scores indicating more severe symptoms (Kaufer et al., 2000). Caregivers completed the NPI-Q as a self-administered survey a few days before the PET scan. The questionnaire covered twelve behavioral and psychological symptoms of dementia, including delusions, hallucinations, agitation/aggression, depression, anxiety, euphoria, apathy, disinhibition, irritability, abnormal motor behavior, and difficulties with eating and sleeping. Based on factor analysis by Aalten et al. (2007), these items were categorized into four domains: (i) hyperactivity (agitation, euphoria, disinhibition, irritability, abnormal motor activity); (ii) psychosis (delusions, hallucinations, nighttime disruptions); (iii) affectivity (depression, anxiety); and (iv) apathy (apathy, eating-related issues).

### 2.4 MRI image acquisition

MRI scans were conducted using a 3.0-T clinical scanner (MAGNETOM Skyra®; Siemens Healthcare, Erlangen, Germany) equipped with a 64-channel phased-array head coil. The imaging protocol included three-dimensional sagittal T1-weighted magnetization-prepared rapid gradient echo acquisition sequences. Parameters were as follows: repetition time = 1800 ms, echo time = 2.92 ms, inversion time = 800 ms, flip angle = 10°, number of excitations = 1, field of view = 240 × 240 mm, acquisition matrix = 256 × 256, voxel size = 0.94 × 0.94 × 1 mm^3^, with 176 contiguous slices.

### 2.5 PET image acquisition and analysis

The procedures for PET image acquisition and analysis have been previously described (Yasuno et al., 2022). Among second-generation TSPO radiotracers known for high target affinity, we selected ^11^C-DPA-713 for TSPO-PET imaging based on two primary considerations. First, in a comparative study involving a blocking agent, DPA-713 exhibited the greatest binding potential in humans, surpassing PK11195, ER176, and PBR28, which suggests an improved signal-to-noise ratio (Fujita et al., 2017). The researchers noted that while DPA-713 radio-metabolites did cross into the brain, their quantification was affected only in individuals with the low-affinity binding genotype, not in those with the high-affinity genotype, which includes all participants in this study. Additionally, a prior study showed that the non-displaceable binding potential of DPA-713 could be reliably measured using the Braak 6 region as a reference area (Yasuno et al., 2022).

Patients underwent 3D PET imaging (Biograph True V; Siemens Healthcare, Erlangen, Germany) within 0–60 min after receiving a bolus intravenous injection of 419.2 ± 21.4 MBq of ^11^C-DPA-713 (range, 382–452 MBq). The mean ± standard deviation (SD) of administered 11C-DPA-713 was 5.8 ± 1.7 nmol (range, 4.2–10.3 nmol). The imaging session was divided into time intervals as follows: 6 × 10 s, 3 × 20 s, 2 × 60 s, 2 × 180 s, and 10 × 300 s. Arterial blood samples were collected manually, with radioactivity concentrations measured 12 times within the first 2 min post-injection, 14 times over the next 28 min, and subsequently every 15 min until the end of the scan. Using these measurements, time-radioactivity curves were generated for whole blood and plasma. Radiometabolite analysis on manually collected arterial blood samples at 5, 15, 30, 45, and 60 min post-injection was performed using a radio-HPLC system (Prominence LC-20 system, Shimadzu, Kyoto, Japan, and FC-4100, Eckert & Ziegler Radiopharma, Hopkinton, MA, USA). Plasma arterial input functions for parent radioactivity were established by adjusting plasma radioactivity based on ligand metabolism data.

PET images were adjusted for scatter, attenuation, and time-of-flight effects, and reconstructed with the ordered subset expectation maximization technique. The resulting images consisted of 109 slices with dimensions of 168 × 168 voxels, each voxel measuring 2.04 × 2.04 × 2.03 mm^3^. Dynamic PET images underwent motion correction and were aligned to the Montreal Neurological Institute (MNI) space using PMOD's View and Registration and Fusion tools (PMOD Technologies, Zurich, Switzerland). Motion correction was applied frame-by-frame via rigid-body registration between sequential frames. PET images were then spatially normalized to MNI stereotactic space, using transformation parameters obtained from individual 3D-T1 MR images, with regions of interest (ROIs) defined according to the Automated Anatomical Labeling (AAL) atlas (Tzourio-Mazoyer et al., 2002).

Regional radioactivity was quantified within ROIs aligned with the neurofibrillary tangle deposition stages outlined by Braak and Braak (1991). Time-activity curves were obtained from ROIs identified in the AAL atlas corresponding to Braak stages 1–3, which include the hippocampus, parahippocampal gyrus, amygdala, lingual gyrus, and fusiform gyrus. Furthermore, ROIs representing Braak stage 4 regions, such as the insular cortex, anterior cingulate cortex, middle cingulate cortex, posterior cingulate cortex, and temporal cortex, were established as target areas where tangle accumulation is expected in individuals with MCI and mild-to-moderate AD.

In addition, a composite ROI encompassing stage 6 regions (such as the precentral gyrus, calcarine cortex, cuneus cortex, postcentral gyrus, and paracentral lobule) was designated as the reference region, serving as a non-target area for ^11^C-DPA-713 in individuals with mild-to-moderate AD (Yasuno et al., 2022).

PET images were processed using the graphical Logan method with a metabolite-corrected plasma input function (Logan et al., 1990). Binding potential (BP_ND_) for each ROI was calculated with the formula (*V*_T_^tissue^ - *V*_T_^ref^)/*V*_T_^ref^, where *V*_T_^tissue^ is the *V*_T_ of the target tissue and *V*_T_^ref^ is the *V*_T_ of the reference tissue. This approach also produced a parametric BP_ND_ image using the same formula.

### 2.6 Statistics

Statistical analyses employed two-tailed tests, with significance determined at p < 0.05/n, where n indicates the number of comparisons, applying the Bonferroni correction. A p-value <0.05 was considered statistically significant if a relationship was assumed. Descriptive and correlation analyses were conducted with SPSS version 26.0, while path analysis was conducted using AMOS version 26.0 (IBM Corp., Armonk, NY, USA). Data distribution was evaluated via Shapiro-Wilk tests, and non-normally distributed data were transformed to approximate a Gaussian distribution using the Box-Cox method.

A stepwise multiple linear regression analysis was first performed to determine predictors of plasma leptin concentration. Plasma leptin concentration was set as the dependent variable, while age, sex, and BMI were included as independent variables.

Next, a partial correlation analysis was conducted to investigate the relationship between plasma leptin levels and regional ^11^C-DPA713-BP_ND_, adjusting for age, sex, ADAS-J cog score, CSF Aβ42/40 ratio, and CSF p-tau 181. After identifying significant correlations between plasma leptin levels and ^11^C-DPA713-BP_ND_ in specific ROIs, a whole-brain voxel-based parametric mapping analysis was performed to further examine the extent of significant regions suggested by the ROI analysis. This analysis was conducted using Statistical Parametric Mapping 12 (SPM12; http://www.fil.ion.ucl.ac.uk/spm/) software. Statistical maps were thresholded at the voxel level with P < 0.01 (uncorrected) and a minimum cluster size of k ≥ 180, based on expected cluster sizes derived from random field theory (Hayasaka and Nichols, 2004). A stepwise multiple linear regression analysis was also applied to identify factors predicting regional ^11^C-DPA713-BP_ND_ within the ROIs highlighted in the previous partial correlation analysis. Independent variables included age, sex, CSF Aβ42/40 ratio, CSF p-tau 181 concentration, and plasma leptin concentration. Additionally, plasma pro-inflammatory cytokine levels (CRP, IL-6, IL-1β, and TNF-α) were included as independent variables to assess their effect on the dependent variable.

Thirdly, stepwise multiple linear regression analysis was performed to identify predictors of ADAS-J cog and NPI-Q scores, representing measures of cognitive function and NPS, respectively. Independent variables comprised age, sex, CSF Aβ42/40 ratio, CSF p-tau 181 concentrations, plasma leptin concentration, plasma pro-inflammatory cytokine levels (CRP, IL-6, IL-1β, and TNF-α), and regional ^11^C-DPA713-BP_ND_ within ROIs identified in the prior partial correlation analysis. If regional ^11^C-DPA713-BP_ND_ significantly predicted NPI-Q scores, Spearman's correlation analysis was then employed to investigate relationships between NPI-Q sub-scores (such as hyperactivity, psychosis, apathy, and affectivity) and regional ^11^C-DPA713-BP_ND_.

Finally, path analysis was employed to estimate the relationships among BMI, plasma leptin concentration, regional ^11^C-DPA713-BP_ND_ within ROIs identified in the earlier analysis, ADAS-J cog scores, and/or NPI-Q scores in patients in the AD continuum. The hypothesis tested was as follows: a) patients with higher BMI would exhibit higher plasma leptin concentration; b) higher plasma leptin concentration would be associated with increased ^11^C-DPA713-BP_ND_, which in turn would affect cognitive ability and/or neuropsychiatric symptoms in patients in the AD continuum. Model fit adequacy was evaluated using seven fit indices: chi-square statistic, goodness-of-fit index (GFI), adjusted goodness-of-fit index (AGFI), normed fit index (NFI), Tucker-Lewis Index (TLI), comparative fit index (CFI), and root mean square error of approximation (RMSEA). The model was considered acceptable if the chi-square statistic was non-significant (p > 0.05), RMSEA was less than 0.08, and GFI, AGFI, NFI, TLI, and CFI values were above 0.90 (Kar et al., 2023). Alternative path connections were assessed and included in the model if they significantly improved the fit, as determined by the chi-square statistic.

## 3 Results

### 3.1 Stepwise multiple linear regression analysis of plasma leptin concentration

**Fig. 1:**
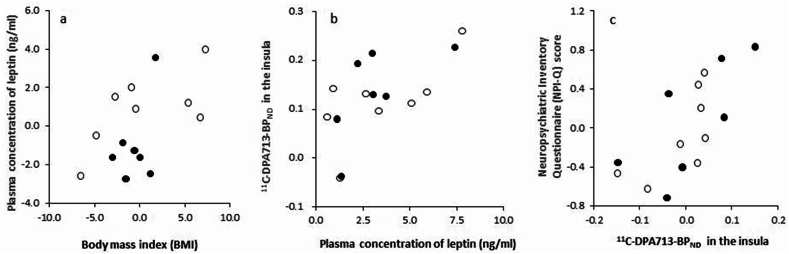
Partial residual plot of plasma concentration of leptin against the body mass index (BMI) (a), ^11^C-DPA713-BP_ND_ in the insula against the plasma concentration of leptin (b) and Neuropsychiatric Inventory Questionnaire (NPI-Q) score against ^11^C-DPA713-BP_ND_ in the insula (c). Filled circle: AD, unfilled circle: MCI. The relation was adjusted for a) sex, b) none, and c) age.

Participant characteristics are outlined in Table 1. The sample included 8 participants with MCI and 7 with clinically diagnosed AD. Descriptive comparisons between these subgroups indicated that participants with AD had slightly lower MMSE scores. Similarly, mean TSPO-PET BP_ND_ values in the insular cortex were modestly higher in the AD subgroup. The comparable medians of NPI-Q between the MCI and AD groups suggest similar central tendencies in NPS; however, the broader upper IQR observed in the AD group indicates that some individuals in this group exhibited substantially higher symptom burden. Table 2 displays the results of the stepwise multiple linear regression analysis predicting plasma leptin levels. The final model included BMI and sex as predictors. A higher BMI (standardized β [sβ] = 0.53, p = 0.04) and being female (sβ = 0.47, p = 0.07) were associated with increased plasma leptin concentration, and the 95 %CI of β value of BMI did not contain 0. Age was excluded from the model. Fig. 1a displays a partial residual plot of plasma leptin concentration against BMI.

**Table 1:** Descriptive characteristics of patients along the AD continuum.

Characteristic/Test	Patients with MCI	Patients with AD	Total AD continuum patients
No.	8	7	15
Sex, M/F	3/5	2/5	5/10
Age, y, mean (SD)	80.5 (4.1)	78.6 (3.6)	79.6 (3.9)
BMI, mean (SD)	21.8 (5.6)	20.7 (1.5)	21.3 (4.1)
ApoE4(+)/(−)	4/4	3/4	7/8
MMSE, mean (SD)	26.3 (2.3)	22.1 (1.2)	24.3 (2.8)
ADAS-J-Cog score, mean (SD)	12.2 (6.3)	11.9 (6.3)	12.0 (6.1)
NPI-Q score, median (IQR)^a^	1.0 (0.0–2.75)	1.0 (0.0–6.0)	1.0 (0.0–3.0)
CSF Aβ42/40 ratio values, median (IQR)^a^	0.061 (0.030–0.068)	0.053 (0.039–0.059)	0.058 (0.037–0.064)
CSF p-tau 181, median (IQR)^a^	42.2 (34.1–79.7)	44.6 (38.1–48.8)	43.4 (36.9–48.8)
Plasma leptin concentration (ng/ml), mean (SD)	3.44 (2.61)	3.12 (2.11)	3.29 (2.31)
Plasma IL-1β concentration (pg/ml), median (IQR)^a^	2.55 (0.63)	3.18 (0.64)	3.18 (1.27)
Plasma IL-6 concentration (pg/ml), median (IQR)^a^	4.03 (2.12)	4.58 (2.19)	4.03 (1.92)
Plasma TNF-α concentration (pg/ml), median (IQR)^a^	6.59 (2.68)	7.11 (1.57)	7.11 (1.05)
Plasma CRP concentration (μg/dl), median (IQR)^a^	6.36 (0.72)	6.53 (0.77)	6.50 (0.74)
Regional^11^C-DPA713-BP_ND_ in Braak 1–3, mean (SD)			
Hippocampal and parahippocampal region	0.03 (0.06)	0.06 (0.13)	0.04 (0.09)
Amygdala	0.16 (0.07)	0.25 (0.13)	0.20 (0.11)
Lingual area	0.17 (0.05)	0.22 (0.06)	0.20 (0.06)
Fusiform area	0.12 (0.05)	0.21 (0.07)	0.16 (0.07)
Regional^11^C-DPA713-BP_ND_ in Braak 4, mean (SD)			
Insular	0.11 (0.08)	0.13 (0.09)	0.12 (0.09)
Anterior cingulum	0.10 (0.07)	0.17 (0.12)	0.13 (0.10)
Middle cingulum	0.23 (0.07)	0.31 (0.09)	0.27 (0.09)
Posterior cingulum	0.15 (0.07)	0.19 (0.11)	0.17 (0.09)

Abbreviations: AD, Alzheimer's disease; MCI, Mild cognitive impairment; SD, standard deviation; BMI, body mass index; ApoE4, Apolipoprotein E4; MMSE, Mini-Mental State Examination; ADAS-J-Cog, Alzheimer's Disease Assessment Scale- Cognitive subscale (Japanese version); NPI-Q, Neuropsychiatric inventory questionnaire; IQR, interquartile range; CSF, cerebrospinal fluid; IL, interleukin; TNF, tumor necrosing factor; CRP, C-reactive protein; BP_ND_, binding potential.

a Non-normal distribution.

**Table 2:** Results of a stepwise multiple linear regression analysis predicting the plasma leptin concentration.

Step	t	Standardized β	β	95 % CI	P	VIF	F	df	p	Adjusted R^2^
Full Model							2.38	3, 11	0.13	0.23
Age	0.34	0.08	0.05	−0.27 to 0.36	0.74	1.02				
Sex	1.93	0.47	2.20	−0.31 to 4.71	0.08	1.05				
BMI	2.12	0.52	0.29	−0.01 to 0.59	0.06	1.07				

Final model							3.79	2, 12	0.05	0.29
Sex	2.02	0.47	2.21	−0.18 to 4.60	0.07	1.05				
BMI	2.28	0.53	0.30	0.01 to 0.58	0.04	1.05				

Abbreviations: CI, confidence interval; VIF, Variance Inflation Factor; R^2^, Multiple regression value squared; BMI, body mass index.

### 3.2 Partial correlation between plasma leptin concentration and regional ^11^C-DPA713-BP_ND_ values

**Fig. 2:**
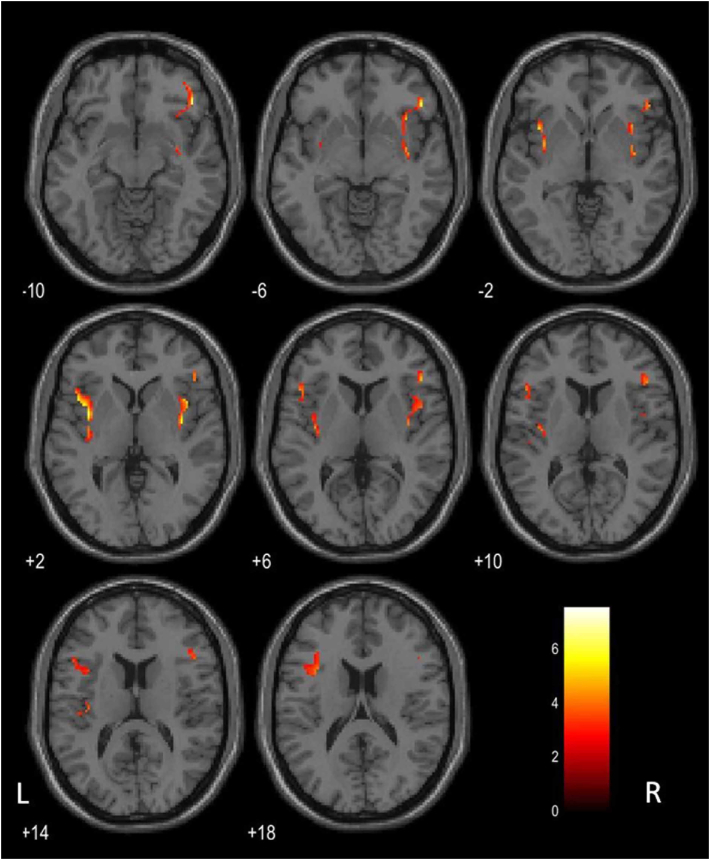
Whole-brain voxel-based parametric mapping analysis to explore the distribution of significant areas within the insula, as indicated by the region of interest analysis. Using partial correlation analysis with age, sex, CSF Aβ42/40 ratio, and CSF p-tau 181 as covariates, positive associations were found between plasma leptin levels and regional ^11^C-DPA713-BP_ND_ values in clusters centered around the bilateral insula and extending anteriorly (p < 0.01).

A partial correlation analysis was conducted between plasma leptin concentration and regional ^11^C-DPA713-BP_ND_ in Braak stages 1–4, adjusting for age, sex, ADAS-J cog score, CSF Aβ42/40 ratio, and CSF p-tau 181 as covariates. Significant associations were found between ^11^C-DPA713-BP_ND_ in the insula and plasma leptin concentration (r = 0.83, p = 0.002) (Table 3, Fig. 1b). In the voxel-based parametric mapping analysis across the whole brain, significant positive associations were identified between plasma leptin levels and regional ^11^C-DPA713-BP_ND_, primarily in clusters within the bilateral insula extending towards the anterior regions (Fig. 2, Table 4).

**Table 3:** Partial correlation analysis between regional^11^C-DPA713-BPND and plasma leptin concentration^a^.

	Correlation coefficients (p value)
Regional^11^C-DPA713-BP_ND_ in Braak 1–3	
Hippocampal and parahippocampal region	0.52 (0.10)
Amygdala	0.24 (0.48)
Lingual area	0.16 (0.63)
Fusiform area	
Regional^11^C-DPA713-BP_ND_ in Braak 4	
Insular	0.83 (0.002)∗
Anterior cingulum	0.44 (0.18)
Middle cingulum	0.44 (0.17)
Posterior cingulum	0.57 (0.07)
Temporal area	0.27 (0.42)

Abbreviations: BP_ND_, binding potential; ADAS-J-Cog, Alzheimer's Disease Assessment Scale- Cognitive subscale (Japanese version); CSF, cerebrospinal fluid.

∗p < 0.00625 (0.05/8).

a Partial correlation analysis with age, sex, ADAS-J cog score, CSF Aβ42/40 ratio values and p-tau 181 concentration was covariated

**Table 4:** Clusters of regions where there was the significant correlation between the plasma leptin concentration and regional^11^C-DPA713-BP_ND_.

Comparison	Brain region	MNI coordinates (x, y, z)^a^	t-value	Cluster size (voxels)
Negative correlation	none			

Positive correlation	Cluster #1: Right insula/inferior frontal area			
	Inferior frontal cortex	48, 32, −8	7.52	343
	Insula	38, −4, 2	5.45	
	Insula	42, 10, 2	4.95	

	Cluster #2: Left insula/inferior frontal area			
	Insula	−36, 0, 0	6.92	327
	Insula	−40, 12, 0	5.55	
	Inferior frontal cortex	−48, 20, 8	4.36	

Abbreviations: BP_ND_, binding potential; MNI, Montreal Neurological Institute.

a x, y, and z reflect coordinates for peak voxel or for other local maxima in MNI space.

Table 5 shows the results of the stepwise multiple linear regression analysis predicting ^11^C-DPA713-BP_ND_ in the insula. The final model included plasma leptin levels, with higher plasma leptin associated with increased ^11^C-DPA713-BP_ND_ in the insula. The 95 % confidence interval for the β coefficient of plasma leptin concentration did not include 0. Other variables were excluded from the final model.

**Table 5:** Results of a stepwise multiple linear regression analysis predicting the^11^C-DPA713-BP_ND_ in the insula.

Step	t	Standardized β	β	95 % CI	P	VIF	F	df	p	Adjusted R^2^
Full Model							1.41	9, 5	0.37	0.21
Age	−1.56	−0.42	−0.01	−0.02 to 0.01	0.17	1.26				
Sex	−0.89	−0.25	−0.04	−0.17 to 0.08	0.42	1.35				
CSF Aβ42/40 ratio values	−0.72	−0.41	−0.01	−0.05 to 0.03	0.51	5.67				
CSF p-tau 181	−0.16	−0.07	−0.10	−2.86 to 2.53	0.88	7.07				
Plasma leptin concentration	2.55	0.94	0.03	0.00 to 0.07	0.05	2.41				
Plasma IL-6 concentration	−0.18	−0.06	−0.08	−1.29 to 1.12	0.87	1.95				
Plasma TNF-α concentration	0.27	0.11	0.007	−0.06 to 0.07	0.80	2.74				
Plasma IL-1β concentration	−0.15	−0.04	−0.02	−0.35 to 0.32	0.89	1.40				
Plasma CRP concentration	0.02	0.01	0.06	−9.46 to 9.58	0.99	2.46				

Final model							9.0	1, 13	0.01	0.36
Plasma leptin concentration	3.00	0.64	0.02	0.01 to 0.04	0.01	1.00				

Abbreviations: BP_ND_, binding potential: CI, confidence interval; VIF, Variance Inflation Factor: R^2^, Multiple regression value squared; CSF, cerebrospinal fluid; IL, interleukin; TNF, tumor necrosing factor; CRP, C-reactive protein.

### 3.3 Stepwise multiple linear regression analysis predicting ADAS-J cog and NPI score

Table 6 provides the outcomes of the stepwise multiple linear regression analysis predicting ADAS-J cog and NPI scores. No significant predictive models emerged for ADAS-J cog scores. However, significant models were identified for predicting NPI scores, with the final model for NPI including ^11^C-DPA713-BP_ND_ in the insula and age as predictors. NPI scores showed a positive association with ^11^C-DPA713-BP_ND_ in the insula (sβ = 0.54, p = 0.004) and a negative association with age (sβ = −0.54, p = 0.004). Fig. 1c displays a partial residual plot of NPI-Q score versus ^11^C-DPA713-BP_ND_ in the insula.

**Table 6:** Results of a stepwise multiple linear regression analysis predicting the ADAS-J-Cog score and NPI-Q score.

Step	t	Standardized β	β	95 % CI	P	VIF	F	df	p	Adjusted R^2^
Model for ADAS**-**J-Cog score										
Full Model							3.29	10, 4	0.130	0.62
Age	−0.11	−0.03	−0.04	−1.03 to 0.95	0.92	1.87				
Sex	−1.11	−0.23	−2.86	−9.99 to 4.28	0.33	1.56				
CSF Aβ42/40 ratio values	−1.89	−0.78	−1.52	−3.75 to 0.71	0.13	6.25				
CSF p-tau 181	−1.23	−0.51	−63.8	−208.3 to 80.8	0.29	7.11				
Plasma leptin concentration	2.38	0.92	2.44	−0.40 to 5.28	0.08	5.54				
Plasma IL-6 concentration	−0.14	−0.03	−3.25	−67.9 to 61.4	0.90	1.96				
Plasma TNF-α concentration	0.92	0.25	1.15	−2.33 to 4.63	0.41	2.78				
Plasma IL-1β concentration	0.36	0.070	2.34	−15.6 to 20.3	0.74	1.41				
Plasma CRP concentration	1.91	0.49	350.8	−158.8 to 860.4	0.13	2.46				
^11^C-DPA713-BP_ND_ in the insula	−1.36	−0.38	−27.00	−72.1 to 18.1	0.21	1.08				
Final model										
none										
Model for NPI-Q score										
Full Model							5.89	10, 4	0.051	0.78
Age	−3.36	−0.58	−0.10	−0.19 to −0.02	0.03	1.87				
Sex	1.99	0.31	0.44	−0.17 to 1.05	0.12	1.56				
CSF Aβ42/40 ratio values	−1.77	−0.56	−0.12	−0.31 to 0.07	0.15	6.25				
CSF p-tau 181	−1.26	−0.42	−5.61	−18.0 to 6.74	0.28	7.11				
Plasma leptin concentration	−0.25	−0.08	−0.02	−0.27 to 0.22	0.81	5.54				
Plasma IL-6 concentration	1.03	0.18	2.05	−3.47 to 7.57	0.36	1.96				
Plasma TNF-α concentration	−2.96	−0.62	−0.32	−0.61 to −0.02	0.04	2.78				
Plasma IL-1β concentration	1.19	0.18	0.66	−0.88 to 2.19	0.30	1.41				
Plasma CRP concentration	1.67	0.33	26.10	−17.4 to 69.6	0.17	2.46				
^11^C-DPA713-BP_ND_ in the insula	3.04	0.72	5.76	0.50 to 11.0	0.04	3.53				

Final model							17.2	2, 12	<0.001	0.70
Age	−3.58	−0.54	−0.10	−0.16 to −0.04	0.004	1.07				
^11^C-DPA713-BP_ND_ in the insula	3.57	0.54	4.33	1.69 to 6.98	0.004	1.07				

Abbreviations: ADAS-J-Cog, Alzheimer's Disease Assessment Scale- Cognitive subscale (Japanese version); NPI-Q, Neuropsychiatric inventory questionnaire; CI, confidence interval; VIF, Variance Inflation Factor: R^2^, Multiple regression value squared; CSF, cerebrospinal fluid; IL, interleukin; TNF, tumor necrosing factor; CRP, C-reactive protein; BP_ND_, binding potential.

Assuming a significant link between any NPI subscore and ^11^C-DPA713-BP_ND_ in the insula, Spearman's correlation analysis indicated significant correlations between ^11^C-DPA713-BP_ND_ in the insula and NPI subscores for psychosis (ρ = 0.64, p = 0.010) and apathy (ρ = 0.61, p = 0.015). The correlations for hyperactivity and depression were ρ = 0.22, p = 0.44 and ρ = 0.32, p = 0.24, respectively.

### 3.4 Path analysis examining the role of leptin as the link between adiposity and NPS in the AD continuum through the development of inflammation in the insula

**Fig. 3:**
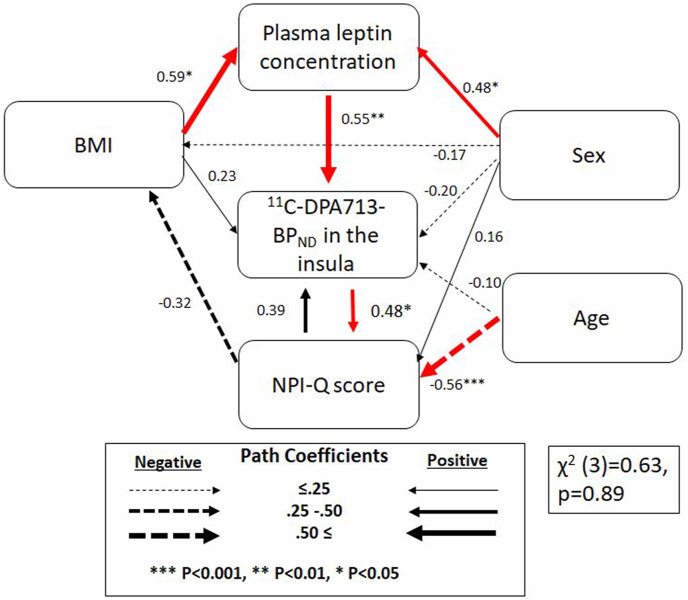
A path analysis model examining the role of leptin as a mediator linking adiposity to neuropsychiatric symptoms via the development of inflammation in the insula. Red color arrows indicate the significant coefficients. Numbers printed next to arrows correspond to standardized regression weights. BMI, body-mass index; NPI-Q, Neuropsychiatric Inventory Questionnaire.

Based on the results, we hypothesized that: a) patients in the AD continuum with higher BMI would exhibit higher plasma leptin concentrations; b) elevated plasma leptin levels would be associated with increased ^11^C-DPA713-BP_ND_ in the insula, which in turn would relate to NPS in the AD continuum. Fig. 3 illustrates the path analysis model of these relationships. Our proposed model, which incorporated additional pathways, including the feedback effect of NPI score on BMI and ^11^C-DPA713-BP_ND_ in the insula, was validated and showed a good fit to the data under the null hypothesis of model-data fit [χ^2^ (3) = 0.63, p = 0.89]. Model fit indices met the required thresholds (CFI = 1.00, GFI = 0.99, AGFI = 0.90, NFI = 0.99, TLI = 1.00, RMSEA = 0.00).

BMI had a significant positive impact on plasma leptin levels (β = 0.59, p = 0.01), which in turn had a significant direct effect on ^11^C-DPA713-BP_ND_ in the insula (β = 0.55, p = 0.03). Additionally, ^11^C-DPA713-BP_ND_ in the insula showed a direct positive relationship with the NPI score (β = 0.48, p = 0.01).

## 4 Discussion

In the regression analysis, plasma concentration of leptin, which was positively related to BMI, was a good predictor of ^11^C-DPA-713 BP_ND_ in the insula in patients in the AD continuum. NPI-Q scores were found to be linked to ^11^C-DPA-713 BP_ND_ in the insula. Significant correlations were identified between the NPI subscores for apathy and psychosis and ^11^C-DPA-713 BP_ND_ in the insula. When we applied path analysis to the data of plasma leptin concentration, as well as data regarding BMI, ^11^C-DPA-713 BP_ND_ in the insula and NPI-Q score, our results supported a role for leptin in linking adiposity to NPS through inflammatory pathways in the AD continuum.

Increased leptin levels associated with adiposity may affect NPS by modulating inflammatory signaling in the insular cortex. Inflammatory processes are crucial in the pathophysiology of the AD continuum, leading to synaptic and circuit dysfunction as well as neurodegenerative changes (Hayley et al., 2021; Teixeira et al., 2008; Van Eldik et al., 2016). Leptin, a hormone mainly produced by adipocytes, plays a crucial role in regulating energy balance and appetite (McGuire and Ishii, 2016). Research has shown that leptin contributes to chronic inflammation, which in turn increases the risk of neurodegenerative diseases in individuals with excess adiposity (Iikuni et al., 2008).

The biological plausibility of our observed association between peripheral leptin levels and regional TSPO expression in the insula may be supported by several mechanistic pathways. Leptin can access the CNS through several pathways: (a) via endothelial cells of the blood-brain barrier (BBB), (b) through epithelial cells in the choroid plexus, and (c) by crossing the basal hypothalamus, where tanycytes form a barrier between the median eminence and the CSF (Ardid-Ruiz et al., 2020; Banks, 2019; Di Spiezio et al., 2018). Once within the central nervous system, leptin acts through its long-form receptor, which is expressed in several brain regions, including pyramidal neurons in the anterior insular cortex (Håkansson et al., 1996; Mercer et al., 1996; Patterson et al., 2011; Shioda et al., 1998). Leptin exhibits proinflammatory properties similar to acute phase reactants and promotes the secretion of proinflammatory cytokines such as TNF-α, IL-6, and IL-1β, via activation of Janus kinase/signal transducer and activator of transcription (JAK/STAT) and mitogen-activated protein kinase (MAPK) signaling pathways (Faggioni et al., 2000; Pérez-Pérez et al., 2020; Shen et al., 2005). These cytokines are key mediators of microglial activation and upregulation of TSPO expression in glial cells (Rupprecht et al., 2010), suggesting that leptin may modulate neuroinflammation in the insula through peripheral-to-central immune signaling mechanisms. These considerations support the proposed pathway linking peripheral metabolic status and central neuroinflammation in the AD continuum, particularly within neural hubs such as the insular cortex, which are implicated in neuropsychiatric symptomatology.

Our findings in patients in the AD continuum indicated that ^11^C-DPA-713 BP_ND_ in the insula cortex is closely related to NPS, specifically psychosis and apathy. Evidence for the insular cortex's dense connectivity, complex interrelationships, central position within the network, and diverse functions suggests that it serves as a pivotal structural hub of the brain (van den Heuvel and Sporns, 2013). The insular cortex shows abnormal gray matter changes in several neurodegenerative and neuropsychiatric disorders (Crossley et al., 2014), and its atrophy may lead to various cognitive impairments and neuropsychiatric conditions. In patients with AD pathology, insular cortex atrophy has been linked to the emergence of neuropsychiatric symptoms such as apathy and psychosis (Rosenberg et al., 2015).

Apathy is a complex state encompassing both an emotional aspect, related to the reward of completing tasks, and a cognitive aspect, associated with initiating and executing actions, leading to reduced motivation for goal-oriented activities (Boublay et al., 2016; Kos et al., 2017). The anterior insula plays a key role in saliency processing, as it is involved in identifying and directing attention to significant stimuli, thereby creating a motivational and goal-driven context for external inputs (Menon and Uddin, 2010). This may help explain the connection between dysfunction in the insular cortex and apathy. Hallucinations and delusions are common psychotic manifestations in AD. Hallucinations are sensory experiences produced by the mind in the absence of external stimuli, whereas delusions are unfounded and irrational beliefs. These symptoms are linked to impaired self-awareness, which affects the ability to recognize that one's perceptions, thoughts, and feelings are manifestations of the illness. The insular cortex is essential for metacognitive self-awareness, as it integrates external sensory inputs with internal state (Menon and Uddin, 2010). Thus, dysfunction of networks related to self-awareness involving the insular cortex may be involved in the development of psychotic symptoms.

We did not observe significant associations between CSF Aβ42/40 ratio or CSF p-tau181 and cognitive performance. This finding may appear unexpected given the well-established roles of these biomarkers in AD pathology; however, it is consistent with previous reports suggesting that their clinical correlations may vary depending on disease stage and timing of measurement. The CSF Aβ42/40 ratio reflects amyloid deposition, which typically plateaus during the symptomatic phase of Alzheimer's disease and may therefore show limited association with current cognitive status (Palmqvist et al., 2015). Similarly, while CSF p-tau181 is a marker of tau pathology, its relationship with cognitive decline may be more prominent in advanced disease or in association with downstream measures of neurodegeneration, such as hippocampal atrophy or synaptic loss (Apostolova et al., 2010; Jack et al., 2010).

In this study, we found that female sex was associated with higher plasma leptin concentrations, while older age was inversely associated with neuropsychiatric symptom severity, as measured by NPI scores. These findings offer further insight into how demographic factors may modulate metabolic and behavioral features along the AD continuum. It is well-established that women exhibit higher circulating leptin levels than men, independent of total adiposity. This sexual dimorphism is attributed to greater subcutaneous fat distribution in females, as well as estrogen-mediated upregulation of leptin gene expression (Saad et al., 1997). Regarding NPS, age emerged as a more robust predictor than sex, with younger participants exhibiting greater NPI scores. This observation is consistent with previous reports that early-onset AD tends to present with more prominent NPS, including affective disturbances, psychosis, and behavioral dysregulation, compared to late-onset cases (Falgàs et al., 2022). Such differences may reflect distinct neuropathological substrates, greater involvement of frontolimbic circuitry, or increased distress and insight in younger patients.

In terms of neuroinflammation, we did not observe significant direct effects of age or sex on TSPO binding in the insular cortex. However, previous studies suggest that TSPO expression may vary with both age and sex. Aging has been associated with increased glial activation and elevated TSPO levels (Schuitemaker et al., 2012). In addition, immune function is known to differ by sex, potentially influencing microglial reactivity and TSPO signal intensity (Laaksonen et al., 2024). The absence of significant age or sex effects in our cohort may reflect the modest sample size or regional specificity of the TSPO signal detected in our PET model.

When interpreting the model as a whole, a nonsignificant path coefficient does not mean that the variable has no effect at all, but rather provides important insights through its connections to other pathways and causal relationships. Although the feedback effects of the NPI score on BMI and ^11^C-DPA713-BP_ND_ in the insula were not significant in our model, they were included based on theoretical and clinical considerations that suggest a bidirectional interaction between neuropsychiatric symptoms, systemic metabolic regulation, and neuroinflammation. Neuropsychiatric symptoms such as apathy, depression, and psychosis are frequently associated with reduced appetite, poor nutritional intake, and unintentional weight loss in patients with AD (White et al., 2024). These behavioral changes may contribute to progressive decreases in BMI, particularly in advanced stages of dementia. Furthermore, chronic psychological stress resulting from persistent NPS may induce sustained activation of the hypothalamic-pituitary-adrenal axis and sympathetic nervous system, leading to increased production of peripheral and central pro-inflammatory mediators (Dantzer et al., 2008). This prolonged inflammatory signaling could, in turn, promote microglial activation and upregulation of TSPO expression in brain regions implicated in stress and affect regulation, such as the insula. While these feedback loops require further investigation using longitudinal or experimental designs, their inclusion in the model reflects the complex and potentially reciprocal nature of interactions between metabolic status, inflammation, and neuropsychiatric burden along the AD continuum.

Importantly, the participants in this study were not obese, as indicated by their relatively low BMI and leptin concentrations. As such, the observed associations between leptin, neuroinflammation, and neuropsychiatric symptoms may not be generalizable to obese populations. Our findings may indicate leptin's role as a pleiotropic metabolic–immune signaling molecule even in non-obese individuals. Excess adiposity is associated with a range of additional pathophysiological mechanisms—including systemic low-grade inflammation, altered adipokine profiles, insulin resistance, and notably, leptin resistance—all of which can modulate both peripheral and central nervous system pathways. In the context of leptin resistance, elevated circulating leptin levels may fail to produce expected satiety, potentially contributing to sustained microglial activation and neuroinflammation. Because our cohort did not include individuals with overt obesity or biochemical evidence of leptin resistance, these complex mechanisms could not be directly assessed. Therefore, we caution against extrapolating our findings to obese populations without further investigation. Future studies should aim to include participants with a broader range of metabolic phenotypes—including those with obesity, insulin resistance, and metabolic syndrome—to clarify the interactions among adipokines, neuroinflammation, and neuropsychiatric symptoms in the AD continuum.

Furthermore, our assessment of inflammation was limited to a focused panel of peripheral markers, and that other inflammatory mediators or downstream neurobiological processes may contribute to the TSPO-PET signal observed in this study. While we included leptin, IL-6, IL-1β, TNF-α, and CRP, we did not assess other cytokines such as MCP-1, IL-18, or markers of microglial activation and phenotype (e.g., CD68 and sTREM2), which may play significant roles in glial reactivity in Alzheimer's disease. Moreover, TSPO expression can be influenced by broader pathophysiological processes triggered by inflammation, including oxidative stress, mitochondrial dysfunction, and disruption of the blood–brain barrier. The current dataset does not allow for a comprehensive evaluation of these mechanisms; therefore, our findings should be interpreted within the scope of the biomarkers assessed. Future studies incorporating a more extensive panel of inflammatory and neuroimmune markers, along with multimodal imaging and longitudinal follow-up, will be essential to elucidate the multifactorial drivers of glial activation and their relevance to neuropsychiatric symptomatology in the AD continuum.

This study has some limitations. First, the current study is based on a relatively small sample size. However, several measures were taken to ensure the appropriateness of the statistical analyses, particularly the path analysis. The use of path analysis in this context was hypothesis-driven, focusing on a limited number of variables with established biological relevance. We applied a simplified model structure to minimize the risk of overfitting and carefully evaluated model performance using multiple fit indices. Notably, all indices—such as the CFI, TLI, and RMSEA—exceeded accepted thresholds, indicating an adequate model fit despite the small sample. Nevertheless, we acknowledge that the small sample size limits the statistical power of the model, and a formal power analysis specific to our PET-based variables was not feasible due to the lack of established effect sizes in this context. Therefore, the current findings should be interpreted as preliminary and hypothesis-generating. Furthermore, the study exclusively involved participants with MCI and mild-to-moderate AD, during which TSPO binding is minimally detected in the neocortical regions. Future studies with larger and more diverse cohorts including patients with more advanced disease are warranted to validate and expand on these findings.

Second, an additional consideration pertains to the diagnostic heterogeneity within our sample. The cohort included individuals with both MCI and clinically diagnosed AD, and this diagnostic diversity likely contributes to the variability in biomarker and behavioral data. To address this, subgroup comparisons between participants with MCI and AD were conducted to better characterize cognitive, behavioral, and biological differences. While the small sample size precludes definitive statistical conclusions, preliminary trends suggest that individuals with AD showed slightly higher TSPO-PET binding and NPI-Q scores, consistent with more advanced disease. These subgroup analyses provide a valuable context for interpreting the observed associations and highlight the importance of considering diagnostic stage when examining the relationships among metabolic, inflammatory, and neuropsychiatric parameters. Future studies with larger samples should further stratify participants by disease stage to better delineate stage-specific mechanisms.

Third, the absence of a healthy control group in our study limits the ability to directly compare the observed levels of plasma leptin and TSPO-PET binding with normative data. However, to contextualize our findings, we reviewed reference values reported in prior studies. Previous reports in healthy elderly populations have shown plasma leptin concentrations ranging from 1.8 to 79.6 ng/mL, with a geometric mean of 12.4, depending on BMI and adiposity (Saad et al., 1997). In our sample, which included relatively lean participants with a mean BMI of 21.3, the average leptin concentration (3.29 ± 2.31 ng/mL) was within or slightly below the expected range. Regarding TSPO-PET binding, previous studies using second-generation tracers have demonstrated significantly lower binding potential in healthy controls compared to individuals with AD (Kreisl et al., 2013; Yasuno et al., 2008, 2022). While these studies provide some comparative context, we acknowledge that inter-study differences in methods, TSPO genotype distribution, and tracer quantification strategies may limit the precision of these comparisons. Inclusion of healthy controls in future studies is necessary to confirm the relative elevation of these biomarkers and further validate our model.

Finally, while our primary hypothesis focused on the role of leptin as a potential upstream modulator of neuroinflammation, it is important to acknowledge that this relationship is neither unidirectional nor isolated. Inflammatory processes can also influence leptin production, particularly through the action of cytokines such as IL-6 and TNF-α, which have been shown to upregulate leptin synthesis in adipose tissue (La Cava and Matarese, 2004). This suggests that leptin and inflammation are engaged in a reciprocal regulatory loop, rather than a one-way pathway. Moreover, the neuroinflammatory state is multifactorial. In addition to leptin dysregulation, several other contributors—including insulin resistance, mitochondrial dysfunction, oxidative stress, proinflammatory adipokines such as MCP-1 and resistin, and gut microbiota-derived endotoxins—are implicated in driving peripheral and central inflammation (Deopurkar et al., 2010; Guillemot-Legris and Muccioli, 2017). Given these complex interactions, the associations observed in this cross-sectional study should not be interpreted as evidence of a direct or causal relationship from leptin to neuroinflammation and neuropsychiatric symptoms. Rather, they likely reflect interdependent processes within a broader network of metabolic and immune dysregulation. To better elucidate these mechanisms, future longitudinal studies incorporating a diverse panel of inflammatory and metabolic markers are needed to clarify causal directions and identify potential therapeutic targets in the AD continuum.

In conclusion, our in vivo findings support the involvement of leptin in mediating the association between adipose-related metabolic dysfunction and neuropsychiatric symptoms through insular inflammation, as illustrated in Fig. 3. The sample size in this study was modest; its findings await confirmation by future research involving larger cohorts. Nevertheless, our findings suggest that elucidating the interplay between leptin, adipose tissue dysfunction, and neuroinflammation is crucial for advancing novel preventive and therapeutic approaches to neuropsychiatric symptoms. The intervention to this interaction, such as maintaining healthy weight and appropriate use of anti-inflammatory drugs, could be the next generation of therapies for NPS in patients in the AD continuum.

## CRediT authorship contribution statement

**Fumihiko Yasuno:** Writing – original draft, Resources, Methodology, Investigation, Funding acquisition, Formal analysis, Conceptualization. **Kazuyuki Nakagome:** Writing – review & editing, Resources, Project administration, Investigation, Funding acquisition. **Yoshie Omachi:** Writing – review & editing, Resources, Investigation. **Yasuyuki Kimura:** Writing – review & editing, Validation, Resources, Methodology, Investigation, Formal analysis, Data curation. **Aya Ogata:** Writing – review & editing, Resources, Investigation. **Hiroshi Ikenuma:** Writing – review & editing, Resources, Investigation. **Junichiro Abe:** Writing – review & editing, Resources, Investigation. **Hiroyuki Minami:** Writing – review & editing, Resources, Investigation. **Takashi Nihashi:** Writing – review & editing, Resources, Investigation. **Kastunori Yokoi:** Writing – review & editing, Resources, Investigation. **Saori Hattori:** Writing – review & editing, Resources, Investigation. **Nobuyoshi Shimoda:** Writing – review & editing, Resources, Investigation. **Kensaku Kasuga:** Writing – review & editing, Resources, Investigation. **Takeshi Ikeuchi:** Writing – review & editing, Resources, Investigation, Funding acquisition. **Akinori Takeda:** Writing – review & editing. **Takashi Sakurai:** Writing – review & editing. **Kengo Ito:** Writing – review & editing. **Takashi Kato:** Writing – review & editing, Project administration.

## Data availability statement

Data not presented in the article will be made available upon request by other investigators for the purpose of replicating procedures and findings.

## Ethics statement

All study procedures received approval from the Institutional Review Board of the National Center for Geriatrics and Gerontology (Approval Nos. 1276 and 20TB9). Informed written consent was obtained from each participant before study participation.

## Funding

This work was supported by the Japan Health Research Promotion Bureau Research Fund (2020-B-07), the Japan Agency for Medical Research and Development (JP23dk0207059 and JP23dm0207073)、and the Japan Society for the Promotion of Science (22K07610), National Center for Geriatrics and Gerontology (22-5, 22–23).

## Declaration of competing interest

The authors declare that they have no known competing financial interests or personal relationships that could have appeared to influence the work reported in this paper.

## Glossary

AD: Alzheimer's disease
Aβ: amyloid-beta
p-tau: tau protein
NPS: neuropsychiatric symptoms
CNS: central nervous system
IL: interleukin
PET: positron emission tomography
TSPO: translocator protein
BMI: body mass index
ADAS-J cog: Alzheimer's Disease Assessment Scale-Japanese Cognitive Subscale
NPI-Q: Neuropsychiatric Inventory Questionnaire
MCI: mild cognitive impairment
CSF: cerebrospinal fluid
MMSE: Mini-Mental State Examination
CDR: Clinical Dementia Rating
EDTA: ethylenediaminetetraacetic acid
CRP: C-reactive protein
MNI: Montreal Neurological Institute
ROI: region of interest
AAL: Automated Anatomical Labeling
BP_ND_: binding potential
TNF-α: tumor necrosis factor-alpha
SD: standard deviation
GFI: goodness-of-fit index
AGFI: adjusted goodness-of-fit index
NFI: normed fit index
TLI: Tucker-Lewis Index
CFI: comparative fit index
RMSEA: root mean square error of approximation
sβ: standardized β
JAK: Janus kinase
STAT: signal transducer and activator of transcription
MAPK: mitogen-activated protein kinase
